# Synthesis, determination, and bio-application in cellular and biomass-bamboo imaging of natural cinnamaldehyde derivatives

**DOI:** 10.3389/fbioe.2022.963128

**Published:** 2022-08-11

**Authors:** Jinlai Yang, Rencong Guo, Huimin Yang, Liangru Wu

**Affiliations:** ^1^ China National Bamboo Research Center, Hangzhou, China; ^2^ Key Laboratory of Bamboo Forest Ecology and Resource Utilization of National Forestry and Grassland Administration, Hangzhou, China; ^3^ Key Laboratory of High Efficient Processing of Bamboo of Zhejiang Province, Hangzhou, China; ^4^ National Longterm Observation and Research Station for Forest Ecosystem in Hangzhou-Jiaxing-Huzhou Plain, Hangzhou, China; ^5^ Bamboo Industry (Jian'ou) Branch, Fujian Provincial Collaborative Innovation Institute, Jian'ou, China

**Keywords:** natural cinnamaldehyde, cinnamaldehyde derivatives, synthesis, determination, bio-application

## Abstract

Cinnamon essential oil (CEO) is the main ingredient in the renewable biomass of cinnamon, which contains natural cinnamaldehyde. To valorize the value of cinnamaldehyde, two simple and useful compounds (**1** and **2**) from CEO were synthesized using a Schiff-base reaction and characterized by infrared spectra (IR), nuclear magnetic resonance (NMR), and high-resolution mass spectrometry (HRMS). Compound **1** was used to confirm the presence of Fe^3+^ and ClO^−^ in solution, as well as compound **2**. Using fluorescence enhancement phenomena, it offered practicable linear relationship of **1**’s fluorescence intensity and Fe^3+^ concentrations: (0–8.0 × 10^−5^ mol/L), *y* = 36.232*x* + 45.054, *R* ^*2*^ = 0.9947, with a limit of detection (LOD) of 0.323 μM, as well as compound **2**. With increasing fluorescence, F_404_/F_426_ of **1** and the ClO^−^ concentration (0–1.0 × 10^−4^ mol/L) also had a linear relationship: *y* = 0.0392*x* + 0.5545, *R* ^*2*^ = 0.9931, LOD = 0.165 μM. However, the fluorescence intensity of **2** (596 nm) was quenched by a reduced concentration of ClO^−^, resulting in a linear. In addition, compounds **1** and **2** were used to image human astrocytoma MG (U-251), brain neuroblastoma (LN-229) cells, and bamboo tissue by adding Fe^3+^ or ClO^−^, with clear intracellular fluorescence. Thus, the two compounds based on CEO could be used to dye cells and bamboo tissues by fluorescence technology.

## Introduction

The main aromatic compound in cinnamon essential oil (CEO) is cinnamaldehyde, with a content of 80%–94.8%, and which can be directly extracted from cinnamon (Liu et al., 2021). This renewable biomass of cinnamaldehyde has many useful functions, such as anti-*Leishmania* activity (Brustolin et al., 2022), antifungal activity (Niu et al., 2022), antibacterial activity (Wang et al., 2021), antimicrobial activity (Thirapanmethee et al., 2021), and improvement of wood decay resistance (Fang et al., 2021). Furthermore, it is easy to synthesize derivatives with biological activity using cinnamaldehyde, such as cinnamaldehyde-based aspirin derivatives (Lu et al., 2018) and chitosan-cinnamaldehyde cross-linked nanoparticles (Gadkari et al., 2019). The Schiff-base fluorescence compounds derived from natural cinnamaldehyde have already been used to sense ClO^−^ and Cu^2+^, and to image U-251 and Hu-7 cells (Yang et al., 2021a). In order to further add the value of natural cinnamaldehyde in the field of fluorescence, the continued synthesis of useful fluorescent compounds from cinnamaldehyde is highly desirable.

As an important microelement in the human body, ferric ion (Fe^3+^) is a vital part of ferrithionein and heme, playing a crucial role in the physiological activities of oxygen delivery, transcription regulation, enzyme catalysis, and metabolism (Huang et al., 2019a; Li S. et al., 2020; Lin et al., 2022). In the human body, levels of endogenous Fe^3+^ that are too high or low can result in heart failure, anemia, and Parkinson’s disease (Wang et al., 2019; Li Y. et al., 2020). In recent years, many novel organic fluorescents used to detect Fe^3+^ have been reported (Song et al., 2019; Rani and John, 2020; Perumal et al., 2021). A fluorescence sensor (**AH2**) was developed to sense Fe^3+^ in aqueous media (Petdum et al., 2021), and a fluorescence chemosensor with a microscale multi-functional metal-organic framework was also used to sense Fe^3+^, as well as Al^3+^ and 2-hydroxy-1-naphthaldehyde (Kang et al., 2016). Interestingly, porous tetraphenylethylene-based organic polymer (**PTOP**) could response Fe^3+^ (turn-off) with high selectivity and sensitivity (Zheng et al., 2020), and a “turn-on” fluorescence sensor (polymer) based on imidazole-functionalized polydiacetylene has also been used to sense Fe^3+^ (Shin et al., 2022). Thus, the synthesis of new, simple, and efficient fluorescence compounds for the determination of Fe^3+^ is of great significance.

As a reactive oxygen species (ROS), hypochlorite anion (ClO^−^) can be obtained by the oxidative reaction of H_2_O_2_ and Cl^−^ (Dong et al., 2020; Pei et al., 2020; Elmas, 2022; Pei et al., 2022), which is widely used in the field of sterilization agents, bleaching agents, and deodorants (Wang et al., 2015; Huang et al., 2016; Dong et al., 2017; Sitanurak et al., 2018; Mahdizadeh et al., 2020). Nevertheless, there is much evidence that excessive generation of ClO^−^ can cause diseases, such as cancer, atherosclerosis, neuron degeneration, cardiovascular disease, lung injuries, and kidney disease (Huang et al., 2016; Zhang et al., 2017; Feng et al., 2018; Song et al., 2018; Ma et al., 2020; Zhang et al., 2020). To date, many good organic fluorescence probes for ClO^−^ have been synthesized, including benzothiazole-based fluorescence and colorimetric chemosensors (Suh et al., 2022), phenanthroimidazole-based fluorescence (Yang et al., 2022), and thiophene-cyanostilbene Schiff-base sensor (Guo et al., 2021). Therefore, it is imperative to design simple sensors for monitoring ClO^−^.

We report two simple Schiff-base derivatives (**1** and **2**) based on cinnamaldehyde, with both compounds **1** and **2** sensitive to the presence of Fe^3+^ and ClO^−^. The optical properties and application potential in bio-imaging of **1** and **2** are systematically investigated for a further study.

## Materials and methods

### Materials and instruments

All reagents (without further purification) were purchased from commercial suppliers. All experiments involving all compounds (**1** and **2**) were conducted in a PBS buffer solution (pH = 7.4, 10 mM, 50% (v/v) C_2_H_5_OH). Fluorescence spectra were obtained using a PerkinElmer LS 55 fluorescence spectrophotometer. UV-vis absorption spectra were measured on a UV-2550 spectrophotometer (SHIMADZU). ^1^H and ^13^C-NMR spectra were determined using a Bruker FT-NMR spectrometer (600 MHz). Infrared spectra were recorded on an FT-IR infrared spectrometer (Nicolet 380). Fluorescence bio-imaging were finished using confocal laser scanning microscopy.

### Synthesis

*Synthesis of 2-amino-3-(3-phenyl-allylideneamino)-but-2-enedinitrile (* ***1*** *).* Cinnamaldehyde (10 mmol), diaminomaleonitrile (10 mmol), and ethanol (50 ml) were added to a 250 ml dried flask with three necks. The contents were then stirred with refluxing for 3.5 h to offer reactant. Using ethanol to recrystallize, a deep-yellow flaked material was produced (57.5%, yield). FT-IR (KBr) ν (cm^−1^): 3,447, 3,287, 3,133, 2,231, 2,205, 1,615, 1,602, 1,584, 1,450, 1,372, 1,308, 1,146, 992, 951, 751; ^1^H NMR (DMSO-*d*_*6*_, 600 MHz): 7.00-7.04 (m, 1H), 7.35-7.38 (t, 4H), 7.40-7.59 (m, 2H), 7.75 (s, 2H), 8.08-8.09 (d, 1H); ^13^C NMR (DMSO-*d*_*6*_, 150 MHz), δ (ppm): 104.15, 114.18, 114.95, 126.72, 127.66, 128.07, 129.47, 130.21, 135.94, 144.46, 157.54; HRMS (m/z): [M + Na]^+^ calcd for C_13_H_10_N_4_+Na^+^, 245.0798; found, 245.0717.

*Synthesis of 2-amino-3-*[*3-(4-dimethylamino-phenyl)-allylideneamino*]*-but-2-enedinitrile (* ***2*** *).* 4-(Dimethylamino) cinnamaldehyde (10 mmol), diaminomaleonitrile (10 mmol), and ethanol (80 ml) were separately added to a 250 ml dried flask (three necks). Then, the contents were stirred with refluxing for 6.5 h to gain reactant. The solution was recrystallized using ethanol, producing a crimson crystal (61.3%, yield). FT-IR (KBr) ν (cm^−1^): 3,450, 3,296, 3,175, 2,909, 2,224, 2,205, 1,661, 1,650, 1,604, 1,583, 1,550, 1,440, 1,367, 1,226, 1,186, 1,145, 992, 812; ^1^H NMR (DMSO-*d*_*6*_, 600 MHz): 2.98 (s, 6H), 6.73-6.75 (t, 2H), 7.28-7.31 (d, 1H), 7.43-7.49 (m, 4H), 7.55-7.57 (m, 1H), 8.02-8.04 (d, 1H); ^13^C NMR (DMSO-*d*_*6*_, 150 MHz), δ (ppm): 104.91, 112.19, 112.44, 122.43, 123.50, 123.74, 125.05, 129.81, 131.11, 145.98, 158.56; HRMS (m/z): [M + H]^+^ calcd for C_15_H_15_N_5_+H^+^, 266.1400; found, 266.1392.

### Cellular imaging

Compounds **1** and **2** were used to image human astrocytoma MG cells (U-251 cells) and human brain neuroblastoma cells (LN-229 cells) using the method of Yang J. et al., 2020.

### Bamboo imaging

The leafless part of fresh bamboo poles with leaves was immersed in a solution (**1** or **2**: 1 × 10^−3^ mol/L) for 1.5 h. After that, it was cut into slices to observe their microstructure with or without adding a drop of Fe^3+^ (or ClO^−^) solution (1 × 10^−3^ mol/L) using an LSM710 confocal fluorescent microscope.

## Results and discussion

### Synthesis

**SCHEME 1:**
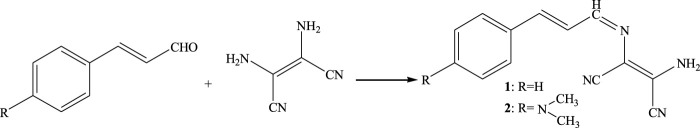
Synthesis of cinnamaldehyde Schiff-base derivatives.

The two derivatives, **1** and **2**, were synthesized using cinnamaldehyde (Scheme 1) (Robertson and Vaughan, 1958) and characterized using IR, NMR, and HRMS. Compounds **1** and **2** were confirmed to be 2-amino-3-(3-phenyl-allylideneamino)-but-2-enedinitrile and 2-amino-3-[3-(4-dimethylamino-phenyl)-allylideneamino]-but-2-enedinitrile.

### Fluorescence spectral response

**FIGURE 1:**
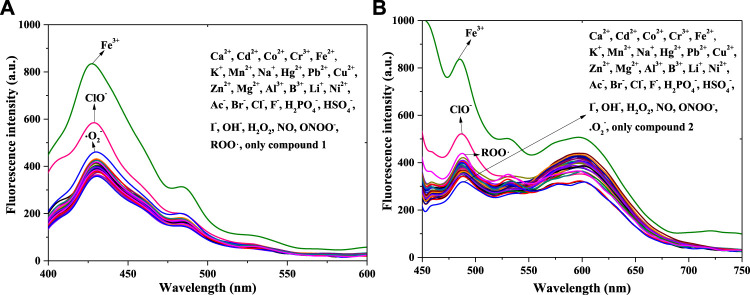
Fluorescence spectra of compound **1 (A)** and compound **2 (B)** with equal positive ions or anions or ROS in a PBS buffer solution: **1**: λ_ex_ = 375 nm, Em. Slit = 5.0 nm, Ex. Slit = 7.0 nm; **2**: λ_ex_ = 430 nm, Em. Slit = 5.0 nm, Ex. Slit = 11.0 nm.

To examine the response of **1** and **2** to the cations Fe^3+^, Ca^2+^, Cd^2+^, Co^2+^, Cr^3+^, Fe^2+^, K^+^, Mn^2+^, Na^+^, Hg^2+^, Pb^2+^, Cu^2+^, Zn^2+^, Mg^2+^, Al^3+^, B^3+^, Li^+^, and Ni^2+^, and to the anions Ac^−^, Br^−^, Cl^−^, F^−^, H_2_PO_4_^−^, HSO_4_^−^, OH^−^, I^−^, ROS of ClO^−^, H_2_O_2_, NO, ONOO^−^, •O_2_^−^, and ROO•, the fluorescence selectivity of compounds **1** and **2** in PBS buffer solution (pH = 7.4, 10 mM, 50% (v/v) C_2_H_5_OH) was studied using fluorescence spectra (concentrations: 1 × 10^−5^ mol/L) (Figure 1).

Figure 1A shows peak fluorescence intensities (400–450 nm**,** compound **1**) with adding equal Fe^3+^ or ClO^−^ had a significant enhance, after adding other substance in compound **1**’s system, the intensity changed little except •O_2_^−^ (weak fluorescence enhancement). Therefore Fe^3+^ and ClO^−^ could response with compound **1,** with a fluorescence enhancement in solution. In contrast with compound **1**’s chemical structure, that of compound **2** has a *p*-N(CH_3_)_2_ in benzene ring; the remainder of the structure is exactly the same as that of compound **1**. Thus, the 32 positive ions, anions, and ROS were separately used in response with compound **2** [Figure 1B]. The addition of Fe^3+^ or ClO^−^ (450–550 nm) also enhanced the fluorescence of compound **2** in the PBS buffer solution. That is, compound **2** also responded to Fe^3+^ and ClO^−^, which is consistent with the results for compound **1**. However, the intensity (550–650 nm) was quenched after adding ClO^−^ in **2**’s solution. The reason for this might be connected with the functional group of *p*-N(CH_3_)_2_. Finally, both compounds **1** and **2** had the potential to detect Fe^3+^ and ClO^−^ in solution.

**FIGURE 2:**
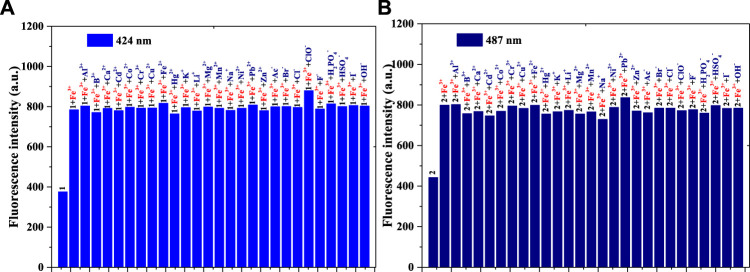
**(A)** Fluorescence intensity (424 nm) of compound **1**, **1** + Fe^3+^, and **1** + Fe^3+^ when adding another ion to the PBS buffer solution: λ_ex_ = 375 nm, Em. Slit = 5.0 nm, Ex. Slit = 6.0 nm; **(B)** Fluorescence intensity (487 nm) of compound **2, 2** + Fe^3+^, and **2** + Fe^3+^ when adding another ion to the PBS buffer solution: λ_ex_ = 430 nm, Em. Slit = 5.0 nm, Ex. Slit = 9.0 nm.

The anti-interference performance of compounds **1** and **2** (1 × 10^−5^ mol/L) to Fe^3+^ (1 × 10^−5^ mol/L) in the same PBS buffer solution was also investigated [Figure 2A (compound **1**) and Figure 2B (compound **2**)]. Figure 2A shows that, compared with those of compound **1**, the other fluorescence intensities increase significantly. Compared with the intensity of **1+**Fe^3+^ **,** after adding another substance to the **1+**Fe^3+^ system, only the solution of **1+**Fe^3+^+ClO^−^ enhanced fluorescence weakly; this might be related to the fluorescence enhancement of ClO^−^ for compound **1**. The **1** + Fe^3+^ combination resulted in good interference capacities with other substances. As shown in Figure 2B, compared with the results of Figure 2A, the **2** + Fe^3+^ also had an anti-interference performance for other ions. In contrast, the addition of ClO^−^ in **2+**Fe^3+^ did not enhance the fluorescence, possibly because of the *p*-N(CH_3_)_2_ group in compound **2**. Both compounds **1** and **2**, therefore, have the potential to be used as fluorescence probes for Fe^3+^.

The anti-interference tests of **1**+ClO^−^ (or **2**+ClO^−^) with the oxidizing agents H_2_O_2_, NO, •O_2_^−^, ONOO^−^, and ROO• are detailed in Supplementary Figures S1A–C. The light-emitting systems of **1**+ClO^−^ and **2**+ClO^−^ differed in terms of the anti-interference performance of other ROS. The addition of another ROS in the solution of **1**+ClO^−^ could not significantly change the fluorescence intensity (424 nm), as well as the **2**+ClO^−^ at 487 nm. However, to add other ROS in the **2**+ClO^−^ system could quench fluorescence at 596 nm.

### Linearity

**FIGURE 3:**
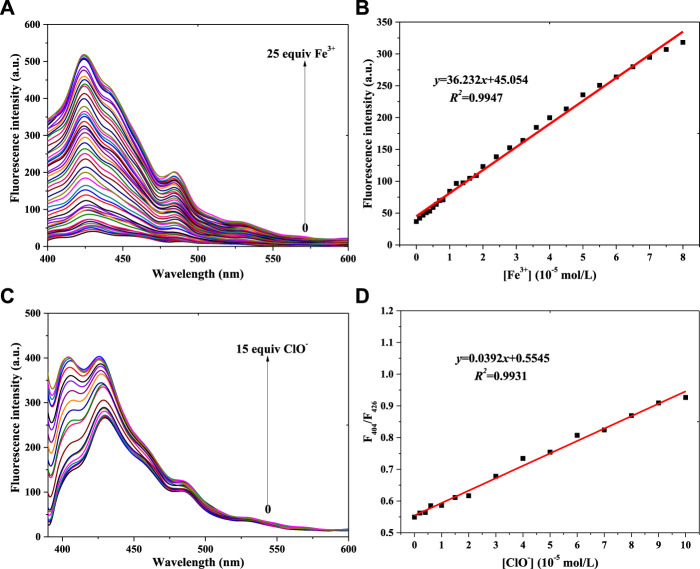
Fluorescence spectra of probe **1** in a PBS buffer solution with various concentrations of Fe^3+^ **(A)** and ClO^−^ **(C)**. **(B)** Linear relationship between the fluorescence intensity of probe **1** and Fe^3+^ concentration. **(D)** Linear relationship between the F_404_/F_426_ of probe **1** and ClO^−^ concentration (λ_ex_ = 375 nm): Fe^3+^: Em. Slit = 5.0 nm, Ex. Slit = 2.5 nm; ClO^−^: Em. Slit = 5.0 nm, Ex. Slit = 5.0 nm.

**FIGURE 4:**
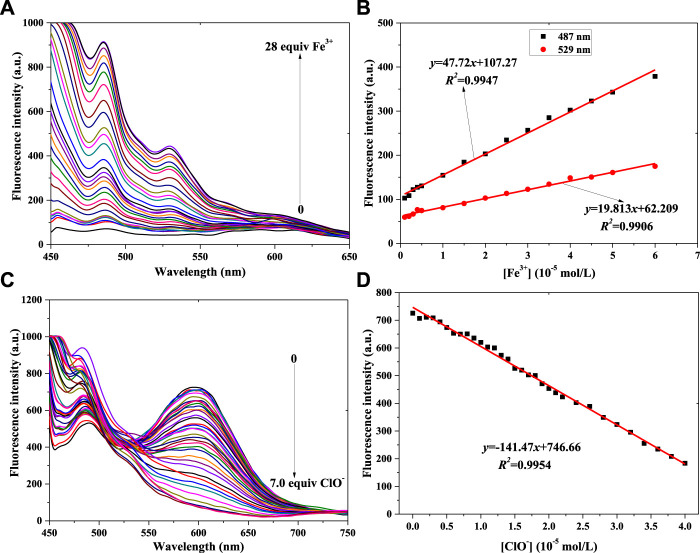
Fluorescence spectra of probe **2** in a PBS buffer solution with various concentrations of Fe^3+^ **(A)** and ClO^−^ **(C)**. Linear relationship between fluorescence intensity of probe **2** and concentration of Fe^3+^ **(B)** and ClO^−^ **(D)** (λ_ex_ = 430 nm): Fe^3+^: Em. Slit = 5.0 nm, Ex. Slit = 5.0 nm; ClO^−^: Em. Slit = 5.0 nm, Ex. Slit = 18.0 nm.

Linearity is very important for fluorescence probes, so the linear relationships between probe **1** or probe **2** and Fe^3+^ and ClO^−^ concentration were investigated in a PBS buffer solution [pH = 7.4, 10 mM, 50% (v/v) C_2_H_5_OH]; the concentration of probe **1** and probe **2** was 1 × 10^−5^ mol/L, and the results are shown in Figure 3 (compound **1**) and Figure 4 (compound **2**).

Figure 3 shows compound **1**’s fluorescence spectra for different concentrations of Fe^3+^ and ClO^−^. Figure 3A shows that the fluorescence intensity (400–450 nm) gradually increases with increasing Fe^3+^ concentration. The peak fluorescence intensity of probe **1** vs. Fe^3+^ concentration is shown in Supplementary Figure S2A. The linear relationship between **1**’s peak intensity and the Fe^3+^ concentration is shown in Figure 3B: *y* = 36.232*x*+45.054, *R* ^*2*^ = 0.9947 (Fe^3+^: 0–8.0 × 10^−5^ mol/L). Using the IUPAC definition of the limit of detection (LOD), an LOD of 0.323 μM was calculated using Eq. 3 *σ*_*bi*_/*m*, which was lower than the 9 μM of a **PDA-Im** sensor (Shin et al., 2022). Fluorescence enhancement might be ascribed to a complexation of **1**’s N atom with Fe^3+^ (Zhou et al., 2017). With the same trend of fluorescence enhancement (**1** + Fe^3+^), the fluorescence intensity was enhanced with increasing ClO^−^ concentration (Figure 3C; Supplementary Figure S2B), with a linear relationship between F_404_/F_426_ and ClO^−^ concentration (Figure 3D). It also showed a good result: *y* = 0.0392*x*+0.5545, *R* ^*2*^ = 0.9931 (ClO^−^: 0–1.0 × 10^−4^ mol/L), with an LOD of 0.165 μM. This LOD value is lower than the 0.238 μM of a coumarin-based fluorescence chemosensor (Elmas, 2022). The reason for **1** monitoring ClO^−^ might be connected with the C=N unit, which reacts with ClO^−^ (Yang Q. et al., 2020).

With a fluorescence enhancement result, compound **2** can be used to sense Fe^3+^. The fluorescence spectra of the **2** + Fe^3+^ solutions are shown in Figure 4A. With the addition of Fe^3+^ from 0 mol/L to 2.8 × 10^−4^ mol/L, the peak fluorescence intensity continuously added (Supplementary Figure S3A). That is, probe **2** might have the same function as probe **1** when helping to determine Fe^3+^ concentration. At the same time, two good linear relationships were obtained, as shown in Figure 4B: 487 nm: *y* = 47.72*x*+107.27, *R* ^*2*^ = 0.9947, LOD = 0.373μM; 529 nm: *y* = 19.813*x*+62.209, *R* ^*2*^ = 0.9906, LOD = 0.451 μM.

Figure 4C shows that the peak intensity at 550–650 nm decreases gradually when ClO^−^ concentration increases from 0 to 6.0 × 10^−5^ mol/L. At higher ClO^−^ concentration, the intensity almost remains constant (Supplementary Figure S3B). The results of adding ClO^−^ to probe **2**’s solution were different from those of adding it to probe **1**’s solution. It might be affected by the *p*-N(CH_3_)_2_ group in probe **2**. Finally, the linear relationship was determined, as shown in Figure 4D. With the concentration range of 0–4.0 × 10^−5^ mol/L (ClO^−^), the peak intensity of **2**’s light-emitting system had a good linear relationship with ClO^−^ concentration: *y* = -141.47*x*+746.66, *R* ^*2*^ = 0.9954, LOD = 0.434 μM.

### UV-vis absorption spectra

**FIGURE 5:**
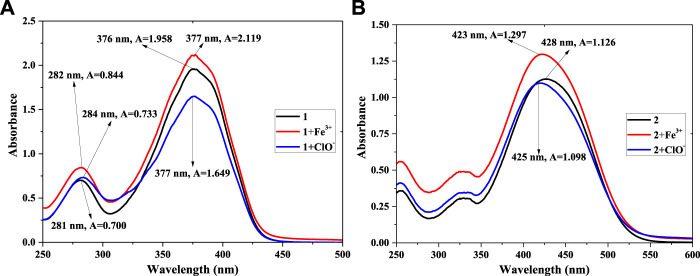
UV-vis absorption spectra of **1, 1** + Fe^3+^, **1**+ClO^−^ **(A)** and **2, 2** + Fe^3+^, **2**+ClO^−^ **(B)** in PBS buffer solution.

To further verify the reaction of **1** (or **2**) and Fe^3+^ or ClO^−^ in PBS buffer solution (pH = 7.4, 10 mM, 50% (v/v) C_2_H_5_OH), UV-vis absorption spectra were recorded. The concentration was 5.0 × 10^−5^ mol/L, as shown in Figure 5. Figure 5A shows a maximum absorption peak (compound **1**) at 376 nm, with A = 1.958. After adding equal amounts of Fe^3+^ ions, the value of A increased to 2.119. Meanwhile, the A value of **1**+ClO^−^ decreased to A = 1.649. This reveals that a chemical reaction had taken place among compound **1** and Fe^3+^ or ClO^−^ in the solution, agreeing with the results shown Figure 1A. In compared with compound **1**, as shown in Figure 5B, the maximum absorption peak of compound **2** red-shifts from 376 to 428 nm, with a decrease in A of 1.126. It might be affected by the *p*-N(CH_3_)_2_ group of **2**. The addition of Fe^3+^ (or ClO^−^) also changed the peak of probe **2**, which had the same trend as probe **1.** The peak of **2** + Fe^3+^ had a weak blue-shift (428–425 nm), and A decreased slightly (1.126–1.098). The reason might also be related to the *p*-N(CH_3_)_2_ group. The results showed that Fe^3+^ and ClO^−^ could response with compounds **1** and **2**, which led to a change in fluorescence intensity in the solution.

### Bio-imaging in live cells

**FIGURE 6:**
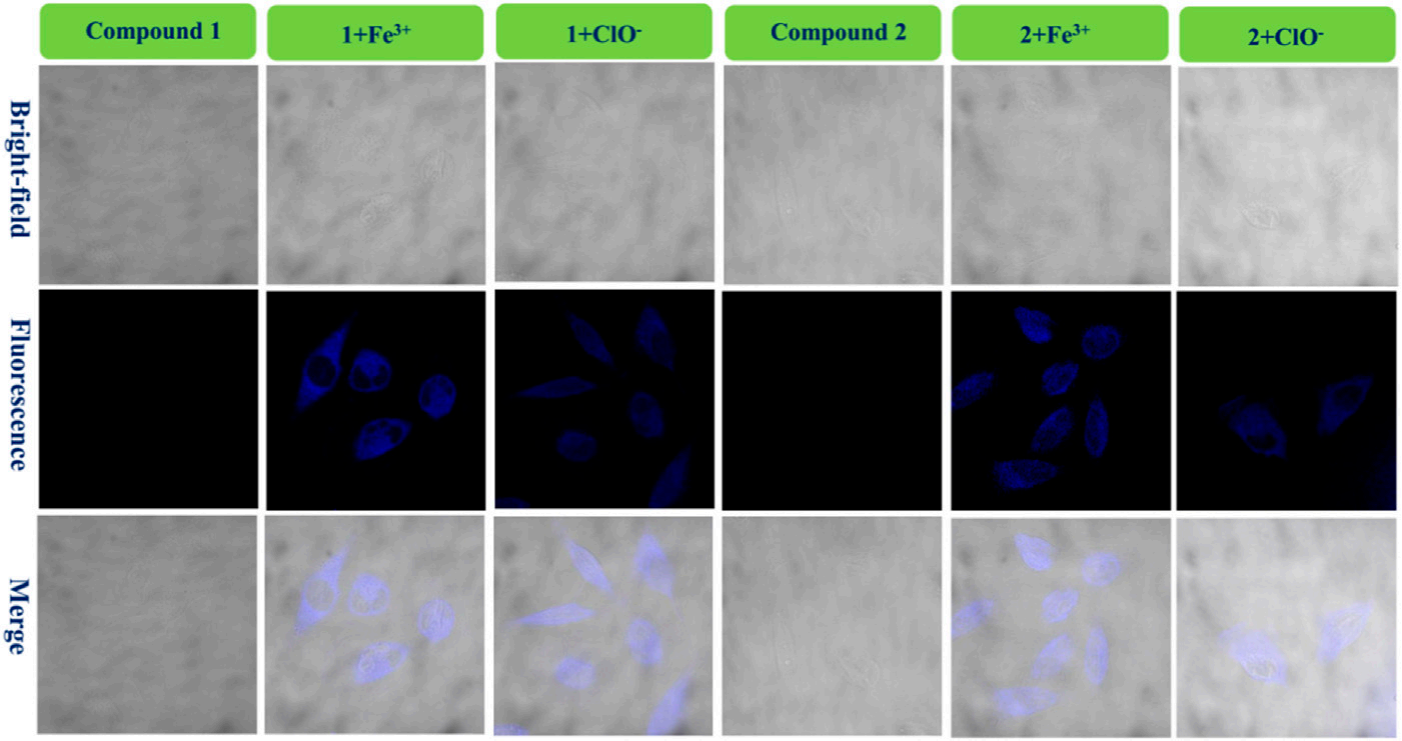
Fluorescence bio-images of the U-251 cells of probe **1**, **1** + Fe^3+^, and **1**+ClO^−^ and probe **2**, **2** + Fe^3+^, and **2**+ClO^−^.

To achieve the value of bio-imaging in live cells (Huang et al., 2019b), probes **1** and **2** (1.0 × 10^−4^ mol/L) were used to dye live U-251 and LN-229 cells (Fe^3+^ or ClO^−^: 1.0 × 10^−3^ mol/L). After dying, confocal fluorescent microscopic images of the cells were taken (ZEISS LSM510) (Figure 6; Supplementary Figure S4).

Figure 6 shows that there is almost no fluorescence for cells dyed only with probe **1** (U-251 cells). However, the U-251 cells produced clear blue fluorescence after adding a drop of Fe^3+^ solution to response with compound **1**. In addition, the **1**+ClO^−^ system had the same function as the **1** + Fe^3+^ system in dying U-251 cells. Therefore, the fluorescence enhancement probe **1** for Fe^3+^ or ClO^−^ could be used in bio-imaging of live U-251 cells. Figure 6 also shows the results for the fluorescence imaging of U-251 cells using compound **2** with the same condition as compound **1**. Probe **2** could sense Fe^3+^ or ClO^−^ in U-251 cells. In order to find the results of imaging in other cells, LN-229 cells were chosen to conduct an experiment—the results are provided in Supplementary Figure S4. Probe **2** led to exactly the same result as probe **1**, in keeping with what is shown in Figure 6. The **1** + Fe^3+^ and **2** + Fe^3+^ permeated the LN-229 cells well and provided bright intracellular fluorescence, as well as the **1**+ClO^−^ and **2**+ClO^−^. The addition of ClO^−^ quenched the intensity of **2**’s fluorescence (550–650 nm) (Figure 4C); however, the **2**+ClO^−^ could also be used to image cells, which might have been connected with the fluorescence at 450–550 nm. Consequently, probes **1** and **2** for Fe^3+^ and ClO^−^ could be used in the bio-imaging of U-251 and LN-229 cells.

### Bio-imaging in bamboo

**FIGURE 7:**
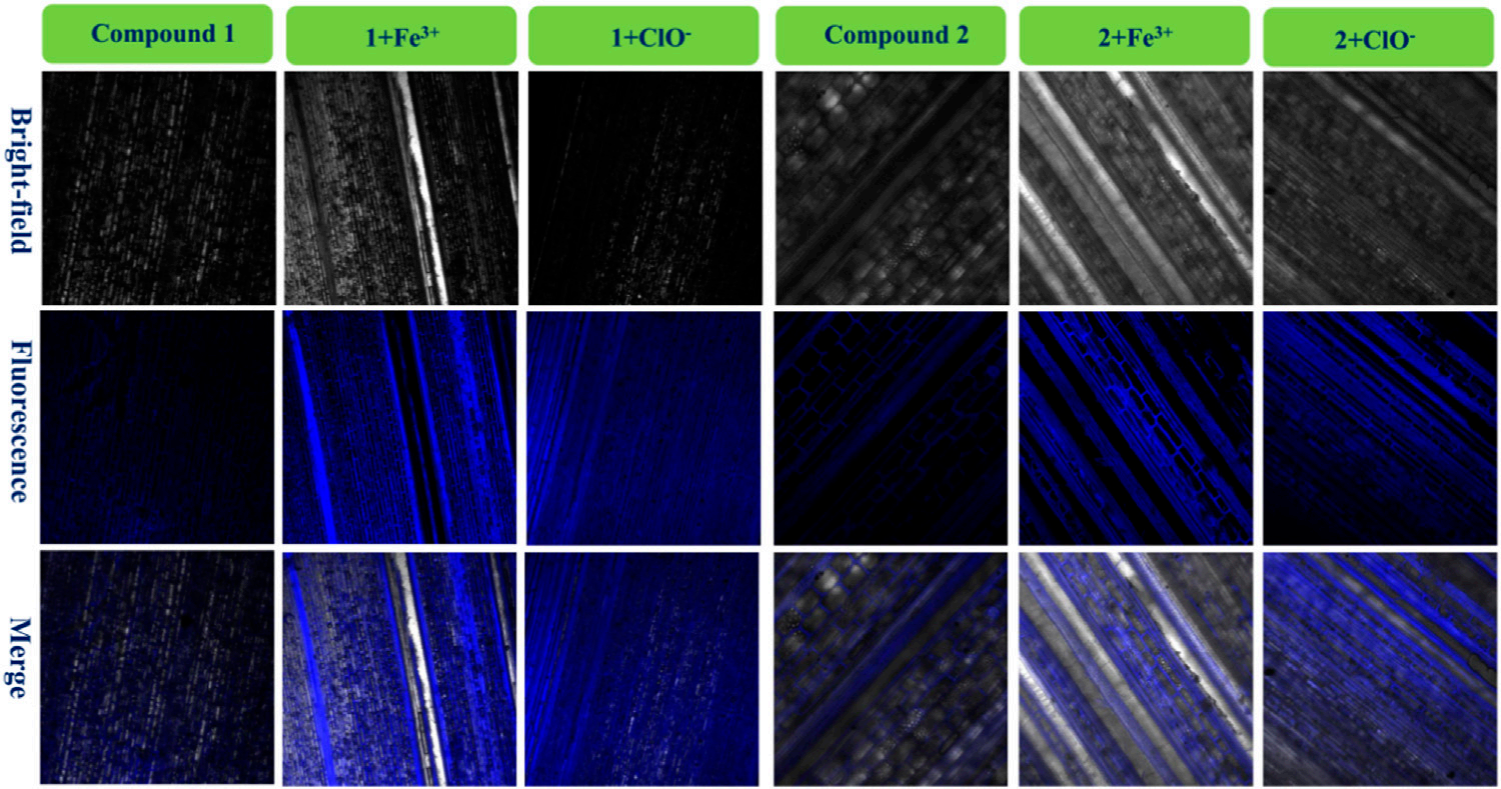
Fluorescence images of bamboo structure using compound **1**, **1** + Fe^3+^, and **1**+ClO^−^ and compound **2**, **2** + Fe^3+^, and **2**+ClO^−^.

Various fluorescence probes can be used to dye plants when required, and bamboo is an important renewable and abundant biomass which can provide wood and shoots (Yang et al., 2021b; Lin et al., 2021; Zheng et al., 2021). In order to investigate the microstructure of bamboo, probes **1** and **2** were used in conjunction with fresh bamboo poles with leaves, the imaging results for **1**, **1** + Fe^3+^, and **1**+ClO^−^ and **2**, **2** + Fe^3+^, and **2**+ClO^−^ are in Figure 7.

It had very weak fluorescence when the bamboo was dyed with **1** or **2**. After adding a drop of the same concentration of Fe^3+^ solution in the bamboo with **1** or **2**, significant blue fluorescence occurred, and the microstructure of the biological tissues was clearly observed. It also told that these fluorescence tissues transferred **1** or **2**. Furthermore, the images of **1**+ClO^−^ and **2**+ClO^−^ were worse than those of **1** + Fe^3+^ and **2** + Fe^3+^, in keeping with Figure 1. Finally, the **1** + Fe^3+^ and **1**+ClO^−^ and **2** + Fe^3+^ and **2**+ClO^−^ could not only dye cells, but could also image the bamboo microstructure.

## Conclusion

Two simple and practical derivatives (**1** and **2**) were synthesized using a chemical reaction of Schiff-base originating from natural cinnamaldehyde and developed for monitoring Fe^3+^ or ClO^−^. Compound **1** could sense Fe^3+^ or ClO^−^ selectively, leading to fluorescence enhancement in a PBS solution, and providing the linear relationship between the fluorescence intensity and the ion concentration. Meanwhile, in compared with compound **1**, probe **2** could also detect Fe^3+^ with increased fluorescence intensity in solution. Nevertheless, the addition of ClO^−^ quenched the fluorescence of **2** at 596 nm. As a result, probe **2** for Fe^3+^ or ClO^−^ also had a favorable linear relation. Finally, compounds **1** and **2** were used in a fluorescence imaging experiment with U-251 cells, LN-229 cells, and bamboo tissues, offering clear intracellular fluorescence with good results. Thus, these two Schiff-base derivatives based on cinnamaldehyde could be used in future fluorescence detection and bio-imaging, adding to the scientific value of the natural biomass of cinnamaldehyde.

## Data Availability

The original contributions presented in the study are included in the article/Supplementary Material, further inquiries can be directed to the corresponding author.
