# Supplementary material for: Synthesis, determination, and bio-application in cellular and biomass-bamboo imaging of natural cinnamaldehyde derivatives
Source: Front Bioeng Biotechnol. 2022 Aug 11;10:963128. doi: 10.3389/fbioe.2022.963128 (PMC9402932; doi:10.3389/fbioe.2022.963128)
Supplement: Supplementary file 1 [file DataSheet1.doc]

**Synthesis, determination, and bio-application in cellular and biomass-bamboo imaging of natural cinnamaldehyde derivatives**

Jinlai Yang^1,2,3,4,5^, Rencong Guo^1,2,3,4^, Huimin Yang^1,2,3,4^, Liangru Wu^1,2,3,4,5*^

**Supplementary Material**

^*^Corresponding author: Prof. Liangru Wu

^1^China National Bamboo Research Center, Hangzhou 310012, Zhejiang, China.

^2^Key Laboratory of Bamboo Forest Ecology and Resource Utilization of National Forestry and Grassland Administration, Hangzhou 310012, Zhejiang, China.

^3^Key Laboratory of High Efficient Processing of Bamboo of Zhejiang Province, Hangzhou 310012, Zhejiang, China.

^4^National Longterm Observation and Research Station for Forest Ecosystem in Hangzhou-Jiaxing-Huzhou Plain, Hangzhou 310012, Zhejiang, China.

^5^Bamboo Industry (Jian'ou) Branch, Fujian Provincial Collaborative Innovation Institute, Jian'ou 353100, Fujian, China.

Email: [bamshoots@163.com](mailto:bamshoots@163.com)

**Figure S1**:

**Figure S1.** (**A**) Fluorescence intensity of compound **1,** **1**+ClO^-^, and **1**+ClO^-^ when adding another ROS to the PBS buffer solution (pH=7.4, 10 mM, 50% (v/v) C_2_H_5_OH), λ_ex_=375 nm, Em. Slit=5.0 nm, Ex. Slit=6.0 nm; (**B**, **C**) Fluorescence intensity of compound **2,** **2**+ClO^-^, and **2**+ClO^-^ when adding another ROS to the PBS buffer solution (pH=7.4, 10 mM, 50% (v/v) C_2_H_5_OH), λ_ex_=430 nm, with Em. Slit=5.0 nm, Ex. Slit=9.0 nm.

**Figure S2**:

**Figure S2**. (**A**) Peak fluorescence intensity of compound **1** *vs.* Fe^3+^ concentrations; (**B**) F_404_/F_426_ of compound **1** *vs.* ClO^-^ concentrations; λ_ex_=375 nm, Fe^3+^: Em. Slit=5.0 nm, Ex. Slit=2.5 nm; ClO^-^: Em. Slit=5.0 nm, Ex. Slit=5.0 nm.

**Figure S3**:

**Figure S3**. Peak fluorescence intensity of compound **2** *vs.* concentrations of Fe^3+^ (**A**) or ClO^-^ (**B**); λ_ex_=430 nm, Fe^3+^: Em. Slit=5.0 nm, Ex. Slit=5.0 nm; ClO^-^: Em. Slit=5.0 nm, Ex. Slit=18.0 nm.

**Figure S4**:


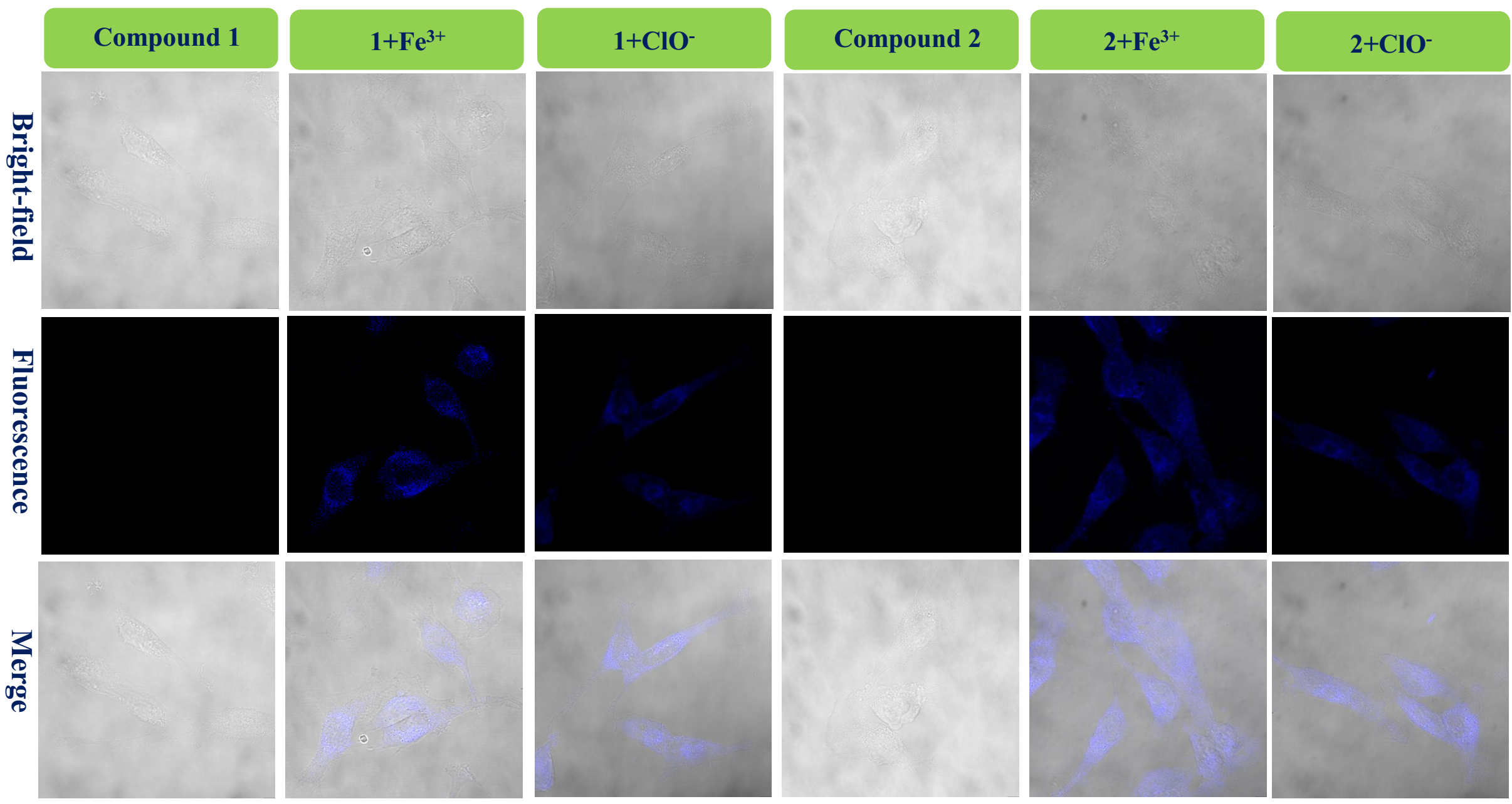


**Figure S4.** Fluorescence bio-images of the LN-229 cells of probe **1**, **1**+Fe^3+^, and **1**+ClO^-^ and probe **2**, **2**+Fe^3+^, and **2**+ClO^-^.
